# Consumer acceptance of genetic-based personalized nutrition in Hungary

**DOI:** 10.1186/s12263-021-00683-7

**Published:** 2021-03-01

**Authors:** Zoltán Szakály, Bence Kovács, Márk Szakály, Dorka T. Nagy-Pető, Péter Popovics, Marietta Kiss

**Affiliations:** 1 Institute of Marketing and Commerce, Faculty of Economics and Business, University of Debrecen, Debrecen, 4032 Hungary; 2 Institute of Applied Economics Sciences, Faculty of Economics and Business, University of Debrecen, Debrecen, 4032 Hungary

**Keywords:** Genetic testing, Personalized nutrition, Consumer acceptance, Psychological processes, Cost and benefit

## Abstract

**Background:**

Despite the increasing number of personalized nutrition services available on the market, nutrigenomics-based level of personalization is still the exception rather than a mainstream activity. This can be partly explained by various factors of consumer acceptance of the new technology. While consumer attitudes toward genetic tests aiming to reveal the risks of a predisposition to various illnesses have already been examined by several research studies worldwide; consumer acceptance of nutrigenomics-based personalized nutrition has only been examined by a significantly lower number of papers, especially in the Central and Eastern European region.

**Objective:**

The purpose of this paper is to examine consumer acceptance of genetic-based personalized nutrition in Hungary. Therefore a national representative survey was conducted involving 1000 individuals. The starting point of the model used is the assumption that the consumer acceptance of personalized nutrition is influenced by its consumer perceptions, which are affected by psychological processes that, in a more general sense, determine acceptance of food innovations.

**Results:**

The results show that 23.5% of respondents accept genetic test-based personalized nutrition. Women were found to reject the new technology in a significantly smaller proportion than men. The relationship between other demographic variables (i.e. age groups, education and subjective income level) and the perception of genetic-based personalized nutrition is also significant. Our results indicate that it is perceived cost/benefit that is most strongly related to genetically based personalized dietary preferences, followed by perceived risk and subjective norms. Perceived uncertainty and perceived behavioural control, however, have only a weak relationship with genetic-based personalized dietary preferences.

**Conclusions:**

Compared with the magnitude of the effect of socio-demographic criteria, it can be concluded that, on the whole, psychological processes in the individual have a greater influence on the development of preferences for genetic-based personalized nutrition than any socio-demographic factor. This also confirms the trend that there are more and more value-added products or value propositions (where a significant part of the value added is to be found in product innovation), for which psychological characteristics are/should be given more emphasis among the segmentation criteria.

**Supplementary Information:**

The online version contains supplementary material available at 10.1186/s12263-021-00683-7.

## Introduction

Currently, non-communicable diseases account for 71% of all global mortality, which amounts to 41 million people a year [1], and according to the forecast of the World Health Organization, these diseases will be attributable to 60% of the burden of disease and 73% of all deaths in 2020 [2]. Chronic diseases, such as obesity, diabetes, cardiovascular diseases and malignant tumours, are imposing an increasing burden on healthcare systems [3–5], while 80% of non-communicable diseases could be prevented by improvements in diet and lifestyle [6]. This raises the question of developing new, more effective strategies for altering dietary habits [3], which may include nutrigenomics-based personalized nutrition [6].

Gene-based personalized nutrition, in other words nutritional genomics or NGx, involves both nutrigenomics, which explores the effects of nutrients on gene expression, and nutrigenetics, which examines the effect of genes on the response to nutrients, i.e. the effect of genetic variation on the interaction between diet and disease [7–12]. Although the distinction between nutrigenomics and nutrigenetics has been made difficult by different and often contradictory definitions and the two terms are often used as synonyms [13], nutrigenomics is the widespread term that covers both aspects of nutritional genomics [14–16].

Genetically based personalized nutrition is based on genetic profiling [17]; thus, the factors of consumer acceptance of genetic tests also appear in the context of nutritional genomics [18]. Consequently, understanding consumer attitudes toward genetic tests is relevant in understanding consumer acceptance of nutritional genomics, as well [17]. Studies on the acceptance of genetic tests indicate positive attitudes on the part of the majority of consumers worldwide (see, e.g. in the USA [18–24]; in the UK [20, 25, 26]; in Australia [27–29]; in the Netherlands [30]; in Sweden [31]; in Spain and China [20]).

In the research studies, the health of the consumers and that of their family members have been identified as key motivators for having genetic testing [19, 21, 22, 25, 27, 28, 32–35]. Those reporting some inherited disease in the family had more favourable attitudes towards undergoing genetic testing [20, 29, 31], and the same holds true for men [23, 25, 26] and the elderly [24, 26]. The effect of education on attitudes towards genetic testing has been mixed according to various studies [36]. Some studies show that those with a lower level of education have more negative attitudes towards genetic testing [37, 38], while others have revealed a reverse relationship [20, 39, 40], or no relationship has been found between the two variables [41, 42].

Previous research studies have suggested that consumers generally have positive attitudes towards nutrigenomic-based personalized nutrition, similar to genetic testing [17, 43–50], although in some research, negative attitudes have dominated [51], and in some cases, the majority of respondents have been hesitant to judge the new technology [52–54]. According to various studies, about one-third to a half of those surveyed would use such a service and adopt a tailor-made, personalized diet [17]; however, considerable differences can be observed between countries. In their research, Stewart-Knox et al. [48] found that in the six European countries surveyed, an average of 66% of respondents would undergo genetic testing and 27% of them would also follow a personalized diet. Of the counties surveyed, it was in Britain and Italy that the highest proportions of respondents would adopt a personalized diet (38.7% and 38.3%, respectively), while the lowest proportion (13.4%) was observed in Germany. However, according to another research study [47], 45% of German consumers would be willing to undergo a nutrigenomic test in order to follow a personalized diet. The results of a research study conducted in Sweden [43] show that 70% of those surveyed would be willing to have a genetic test made in order to receive personalized nutritional advice, 65% would consider following the advice, while 20% would only be willing to do so if some serious illnesses could be prevented in that way. A Hungarian research study [52–54] concluded that only 27% of respondents would adopt nutrigenomics-based personalized nutrition, while about 29% would clearly reject it, and almost half (44%) were indifferent to the new technology.

As regards demographic factors, women are more likely to undergo nutrigenomic tests [47] and be interested in nutrigenomics-based personalized nutritional advice [48]; however, the results are mixed, and depend on age and the existence of health problems. Research studies conducted by Ahlgren et al. [43] and Szakály et al. [52–54] have found younger people more willing to have a nutrigenomic test performed and adopt nutrigenomics-based personalized nutrition, while according to Stewart-Knox et al. [48], it is older people who are more willing to use these services; Roosen et al. [47], however, found no connection between age and willingness to undergo nutrigenomic tests. Furthermore, those aware of their various health problems would prefer to buy functional foods that meet their genetic risks [47] and would take advantage of personalized nutritional advice [48]. In contrast, Ahlgren et al. [43] found no correlation between any health problem and willingness, although 20% of respondents claimed that their willingness would depend on whether or not following the personalized advice would result in developing serious diseases. This implies that the known risk of a disease may motivate respondents to follow advice on such a diet. In terms of income, those with higher income are more receptive to personalized nutrition and would be willing to pay more for such services than those with lower income [52–55]. Finally, the findings of some research studies [52–54] suggest that a higher level of education is accompanied by a more extensive acceptance of nutrigenomics-based personalized nutrition, while other results indicate that education is not related to the willingness to use nutrigenetics and personalized nutrition [47].

Besides demographic factors, several psychological processes have been identified in the literature that influence consumer acceptance of genetic-based personalized nutrition. A fundamental trade-off between the benefits and the privacy risk of nutrigenomics-based personalized nutrition has been revealed by a number of research studies: the higher the consumer benefit because of the more personalized recommendations received, the higher the perceived privacy risk due to the transmission of more and more sensitive information [44, 51, 56–58]. Several studies have also confirmed consumers’ fear of high costs of genetic tests and personalized diets [25, 46, 49, 59], which can significantly hinder acceptance of the new technology. Moreover, consumers’ food choices are largely determined by collective values and social norms [60]; therefore, acceptance of personalized nutrition largely depends on the preferences of their family, friends and other opinion leaders [4, 50, 61]. A particularly important factor in the acceptance of genetic-based personalized nutrition is if the consumer has freedom of choice in terms of undergoing nutrigenomic testing [61, 62]. With this, consumers have greater responsibility toward developing their own health [8, 63], which seems to be appealing to them [43].

Based on the results of previous research studies outlined above, the purpose of this paper is to examine the psychological processes and demographic factors influencing consumer acceptance of genetically based personalized nutrition in Hungary.

## Materials and methods

### Sampling method

Primary data collection was carried out in November 2019 by means of personal interviews conducted at the respondents’ homes. The nation-wide questionnaire-based survey was representative of gender (*χ*^2^(1)=0.760; *p* = 0.383) and age group (*χ*^2^(5)=0.421; *p* = 0.520). In the sampling process, representativeness was also ensured for regions (*χ*^2^(6)=6,997; *p* = 0.321) and settlement types (*χ*^2^(2)=3.409; *p* = 0.182), so the sample structure perfectly matched the quota set in advance by the Hungarian Central Statistical Office (quota sampling).

In each region and selected settlement stratified random sampling was used and the strata variable was the birthday date. This method involves providing each interviewer with a randomly selected starting address in the settlement chosen. From the starting address, in ascending order by house number, the interviewers began the questioning at the third house on the same side of the street, and then, having completed there, they continued at the next third house. When drawing up the sampling plan, it was also made sure that conducting the questioning in a district with detached houses or doing so in a district with blocks of flats should cause no confusion to the interviewers.

During the questioning, first, the interviewer inquired about the number of people aged 18 or above living in the household. Then, the second step was to select the person whose date of birth (birthday) fell closest to the day of the interview from among the family members of the proper age (simply put: who held his/her birthday last). With this method, randomness was ensured in each stratum. In the next step, the questions were read out by the interviewers, to which oral answers were given by the respondents. While doing so, the person selected was handed a so-called set of cards that included the possible answers to each question and the appropriate answers were chosen by the respondent from these options. The interview took a maximum of 15 min for each respondent. The interviewers conducting the survey were monitored with randomly selected telephone calls and also with visitor cards left at the respondent.

The cleaned sample consists of 1000 items. Since in Hungary the number of people in the age group examined (i.e. above 18 years of age) is approximately 8 million [64], with a 95% confidence level and a 5% margin of error, on the basis of Gill and Johnson [65], the required sample size is 385 people; consequently, the sample size is appropriate for our research objectives. Table 1 shows the percentage distribution of socio-demographic groups of individuals involved in the survey and the population composition according to the previously mentioned four factors.

**Table 1:** Distribution of the sample according to background variables (*N* = 1000), population composition according to representative variables

Label	Sample distribution		Population distribution^**1**^
	Count	%	%
Male	471	47.1	47.8
Female	529	52.9	52.2
18–29 years	169	16.9	17.2
30–39 years	161	16.1	16.0
40–49 years	196	19.6	19.6
50–59 years	152	15.2	15.1
60–69 years	163	16.3	16.3
70–years	159	15.9	15.8
Budapest	181	18.1	17.9
Other town	550	55.0	52.6
Village	269	26.9	29.5
Western Transdanubia	100	10.0	10.1
Central Transdanubia	109	10.9	10.8
Southern Transdanubia	94	9.4	9.0
Northern Great Plain	148	14.8	14.8
Central Hungary	298	29.8	31.0
Northern Hungary	119	11.9	11.5
Southern Great Plain	132	13.2	12.7
Primary school	109	10.9	
Vocational school	394	39.4	
High school	364	36.4	
Higher education	133	13.3	
Can live on it very well and can also save	78	7.8	
Can live on it but can save little	392	39.2	
Just enough to live on but cannot save	427	42.7	
Sometimes cannot make ends meet	74	7.4	
Have regular financial problems	9	0.9	
Not known / No answer	20	2.0	

^1^Source of data: [64, 66]

### Structure of the questionnaire

**Fig. 1:**
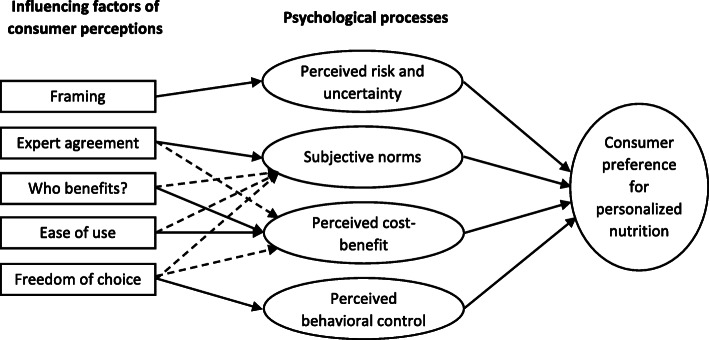
Model of influencing factors of consumer preference for personalized nutrition. Source: Modified from [61]. Dashed arrows indicate relationships additional to the original theoretical model revealed by the empirical analysis

Of the models in the literature that include a comprehensive framework of factors influencing consumer acceptance of genetically based personalized nutrition [16, 44, 56, 61, 67], our research was conducted based on the model developed by Ronteltap et al. [61]. The starting point of this model is the assumption that the consumer acceptance of personalized nutrition is influenced by consumer perceptions of personalized nutrition, which are affected by psychological processes that, in a more general sense, determine acceptance of food innovations (see Fig. 1). These psychological processes consist of perceived risk and uncertainty, subjective norms (i.e. whether significant others are likely to endorse the behaviour), perceived cost-benefits and perceived behavioural control (i.e. whether a person believes he or she can actually perform the behaviour necessary for acceptance). Consumer perceptions include framing (i.e. the positive or negative wording of a message), expected to be mediated by perceived risk and uncertainty; expert agreement on the feasibility and desirability of personalized nutrition, mediated by a directive and positive subjective norm; beneficiaries of personalized nutrition (i.e. the consumer, the science or the industry) and ease of use (i.e. the extent to which personalized diets are perceived as being compatible with current food habits and easy to implement in consumers’ existing lifestyles), both mediated by the perceived cost-benefit ratio; and finally, freedom of choice (i.e. the extent to which consumers are free to choose whether or not to make their genetic profile available), expected to be mediated by perceived behavioural control. In the final empirical model, framing did not contribute significantly to explaining consumer preference for personalized nutrition, but the authors found the following mediation effects of psychological processes in addition to the mediation effects expressed by the theoretical model: perceived cost-benefit ratio mediated expert agreement, subjective norms also played a mediating role when the consumer was the main beneficiary, ease of use and freedom of choice were also mediated by subjective norms, and finally, perceived cost-benefit considerations also proved to be a mediator of freedom of choice in the final model [54, 61].

Before presenting the questions of the questionnaire for measuring model constructions, the interviewers gave the respondents a description of the concept developed by Stewart-Knox et al. [48] (p. 984) on genetic-based personalized nutrition: “In future it may be possible to assess your risk of developing late-onset (type 2) diabetes or heart disease with a genetic test of your saliva (by means of a mouth swab). This may allow you to eat foods that are suitable for your genetic profile. This is genetic test-based personalized nutrition.” First, consumer preference for the development outlined in the previous description was asked (particularly attractive, both attractive and not attractive, not attractive at all). To assess the concepts of the model developed by Ronteltap et al. [61], 16 closed-end questions were used. Among them, influencing factors of consumer perception of personalized nutrition were assessed by 11 statements measured on 5-point Likert-scale, and psychological processes were assessed by 5 dichotomous statements (agree, disagree), modified and adapted from Ronteltap et al. [61], respectively. For the items measuring the model constructs, see Supplementary information, Additional file 1. Socio-demographic background variables such as gender, age, education and subjective income were placed at the end of the questionnaire.

### Mathematical and statistical evaluation

The data processing and modelling methodology of the current research was developed on the basis of Szakály et al. [53] and Ronteltap et al. [61]. Namely, multinominal logistic regression was used to determine the extent to which an individual’s characteristics and distinguishing criteria increase the likelihood of acceptance of a genetically based personalized diet. However, we encountered several problems during modelling: variables that based on the chi-square test clearly significantly influence preferences for personalized nutrition fell from the model (this was presumably due to multicollinearity on the one hand, which is also evidenced by the high VIF index, and to the effort to maximize the explanatory power of the model in multivariate modelling on the other); the combined explanatory power of the model can be regarded as so low (Naglerke *R*^2^ = 0.30) that the interpretation of the results and the determination of the actual correlations became questionable. For these reasons, we decided to report the results of the cross-tabulation analysis and the non-parametric chi-square test, as well as the value of the Cramer’s V factor measuring the power of the effect. This decision has two consequences: (1) the empirical model of the research cannot thus be interpreted as a multivariate model, i.e. the combined effect of the explanatory variables is not examined; (2) all the distinguishing criteria that influence the evolution of an individual’s preferences for genetic-based personalized nutrition can be presented.

## Results

As shown in Table 2, 23.5% of respondents accept genetic test-based personalized nutrition and see it as an attractive option, while nearly a third (30.6%) clearly reject it. The largest group of respondents (45.9%) consists of consumers uncertain about the new technology.

**Table 2:** Preferences for personalized nutrition

I think that a genetic test-based personalized nutrition is…	***N***	Percent
a particularly attractive option; therefore, I would use it.	235	23.5
both attractive and not attractive, with a view to preserving my health.	459	45.9
not an attractive option at all; therefore, I would not use it.	306	30.6
Total	1000	100.0

Source: Authors’ own compilation

**Fig. 2:**
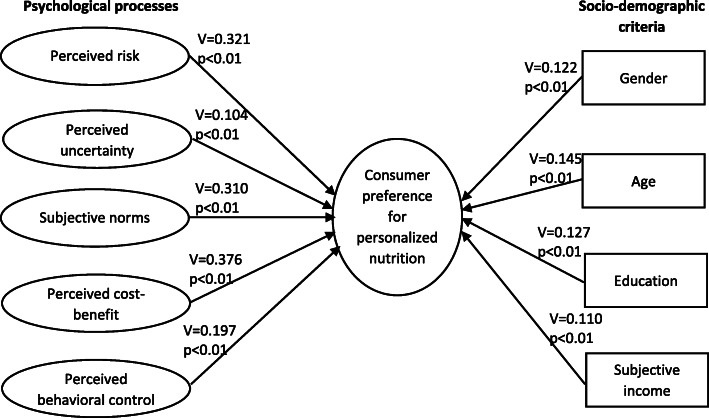
The empirical model of the research. Source: Authors’ own construction

From a practical point of view, the question arises as to who these consumers are, i.e. what socio-demographics they can be described by, what psychological characteristics they have and also what factors determine their preferences for genetic-based personalized nutrition. The current paper intends to report and present only those distinguishing criteria for which there is a significant difference in gene-based personalized dietary preferences. Figure 2 shows the significance levels of the nonparametric chi-square tests as well as the Cramer’s *V* factor values measuring the effect size.

### Socio-demographic characteristics in relation to genetic-based personalized dietary preferences

The socio-demographic characteristics examined in our research were as follows: gender, age, education, subjective income. Based on these characteristics, we found some significant differences in genetic-based personalized nutrition preferences (Table 3).

**Table 3:** Preferences for genetic-based personalized nutrition by psychological characteristics

	Particularly attractive, %	Both attractive and not attractive, %	Not attractive at all, %	***χ***^**2**^	***N***
Gender					
Male	18.9 (− 3.2)	45.6 (− 0.2)	35.5 (3.1)	14.906**	1000
Female	27.6 (3.2)	46.1 (0.2)	26.3 (− 3.1)		
Age					
18–29	24.9 (0.5)	49.7 (1.1)	25.4 (− 1.6)	42.088***	999
30–39	24.8 (0.5)	49.1 (0.9)	26.1 (− 1.4)		
40–49	21.9 (− 0.5)	56.1 (3.2)	21.9 (− 2.9)		
50–59	27.2 (1.2)	44.4 (− 0.4)	28.5 (− 0.6)		
60–69	22.1 (− 0.4)	42.9 (− 0.8)	35.0 (1.3)		
70–	20.1 (− 1.1)	30.8 (− 4.2)	49.1 (5.5)		
Education					
Primary school	14.7 (− 2.3)	42.2 (− 0.8)	43.1 (3.0)	32.408***	999
Vocational school	19.6 (− 2.4)	48.1 (1.1)	32.3 (1.0)		
High school	25.5 (1.1)	44.5 (− 0.7)	29.9 (− 0.3)		
Higher education	36.8 (3.9)	46.6 (0.2)	16.5 (− 3.8)		
Subjective income					
Can live on it very well and can also save	27.3 (0.7)	45.5 (0.0)	27.3 (− 0.7)	22.483**	979
Can live on it but can save little	28.6 (2.9)	46.4 (0.4)	25.0 (− 3.1)		
Just enough to live on but cannot save	19.7 (− 2.7)	46.8 (0.7)	33.5 (1.7)		
Sometimes cannot make ends meet	20.3 (− 0.7)	35.1 (− 1.9)	44.6 (2.7)		
Have regular financial problems	11.1 (− 0.9)	33.3 (− 0.7)	55.6 (1.6)		

Source: Authors’ own compilation. Adjusted standardized residuals are in brackets. **p < 0.01, ***p < 0.001

Women were found to reject the new technology in a significantly (*χ*^2^(2) = 14.906, *p* = 0.001) smaller proportion than men (26.3%, and 35.5%, respectively); in addition more women (27.6%) than men (18.9%) consider the use of genetic test-based personalized nutrition an attractive option. However, gender proved to be a variable with very poor predictive power in terms of genetically based personalized nutrition (Goodman–Kruskal tau = 0.006, *p* = 0.002; uncertainty coefficient = 0.007, *p* = 0.001), and the relationship between the two variables is particularly weak (Cramer’s *V* = 0.122, *p* = 0.001).

The relationship between age groups and the perception of genetic-based personalized nutrition is also significant (*χ*^2^(10) = 42.088, *p* < 0.001). Those over 70 years of age reject genetically based personalized nutrition significantly more than expected (49.1%), while respondents aged between 40 and 49 reject it in a smaller proportion than expected (21.9%). However, age is also a weak predictor in this case (Goodman–Kruskal tau = 0.023, *p* < 0.001; uncertainty coefficient = 0.019, *p* < 0.001), and the relationship between the two variables is weak (Cramer’s *V* = 0.145, *p* < 0.001).

Significant (*χ*^2^(6) = 32.408, *p* < 0.001) differences were found in the acceptance of genetic-based personalized nutrition according to education as well. Those with lower education find personalized nutrition attractive in a significantly lower proportion than expected (primary school (i.e. finished school at 14): 14.7%, vocational training: 19.6%) and reject it in a significantly higher proportion (primary school: 43.1%). Conversely, those with a tertiary education find it more attractive than expected (36.8%) and reject it less than expected (16.5%). Nevertheless, education is also a variable with poor predictive power (Goodman–Kruskal tau = 0.014, *p* < 0.001; uncertainty coefficient = 0.015, *p* < 0.001), which is also confirmed by Cramer’s *V* (0.127, *p* < 0.001).

As regards subjective income, a significant difference (*χ*^2^(8)=22.483, *p* = 0.004) could be observed in the preference for genetic-based personalized nutrition. Those who can live on their income and can also save a little find this option attractive in a significantly higher proportion than expected (28.6%) and reject it in a lower proportion (25.0%). In contrast, those whose income is just enough to live on consider it to be attractive in a lower proportion than expected (19.7%), while those struggling with subsistence problems reject it in a higher proportion (44.6%) than expected. Subjective income, however, is a poor predictor (Goodman–Kruskal tau = 0.011, *p* = 0.008; uncertainty coefficient = 0.011, *p* = 0.005), and the relationship between the two variables is weak (Cramer’s *V* = 0.107, *p* = 0.004).

### Psychological characteristics influencing genetic-based personalized dietary preferences

In our model based on Ronteltap et al. [61], the following psychological characteristics were incorporated: perceived risk, perceived uncertainty, subjective norms, perceived cost and benefit and perceived behavioural control. Based on these characteristics some significant differences in genetic-based personalized dietary preferences were found (Table 4).

**Table 4:** Preferences for genetic-based personalized nutrition by psychological characteristics

	Total, %	Particularly attractive, %	Both attractive and not attractive, %	Not attractive at all, %	***χ***^**2**^	***N***
Perceived risk						
Agree	46.5	13.8 (− 6.8)	41.4 (-2.3)	44.8 (8.9)	93.018***	903
Disagree	53.5	33.3 (6.8)	49.1 (2.3)	17.6 (− 8.9)		
Perceived uncertainty						
Agree	78.8	21.4 (− 2.8)	46.2 (0.0)	32.4 (2.5)	10.279**	955
Disagree	21.2	30.7 (2.8)	46.0 (0.0)	23.3 (-2.5)		
Subjective norms						
Agree	45.0	37.3 (8.0)	42.5 (− 0.8)	20.2 (− 6.5)	78.162***	815
Disagree	55.0	13.2 (8.0)	45.3 (0.8)	41.5 (6.5)		
Perceived costs/benefits						
Agree	55.8	34.9 (7.9)	48.9 (2.8)	16.2 (− 10.5)	127.687***	905
Disagree	44.2	12.0 (− 7.9)	39.5 (− 2.8)	48.5 (10.5)		
Perceived behavioural control						
Agree	63.4	29.9 (5.2)	44.3 (− 0.2)	25.7 (− 4.6)	35.234***	907
Disagree	36.6	14.5 (− 5.2)	45.2 (0.2)	40.4 (4.6)		

Source: Authors’ own compilation. Adjusted standardized residuals are in brackets. **p < 0.01, ***p < 0.001

Our results indicate that 46.5% of respondents believe that a genetically based personalized diet involves many risks (perceived risk). This is moderately strongly related to preference for genetic-based personalized nutrition (*χ*^2^(2) = 93.018; *p* < 0.001; Cramer’s *V* = 0.321, *p* < 0.001), but with poor predictive power (Goodman–Kruskal tau = 0.046, *p* < 0.001; uncertainty coefficient = 0.050, *p* < 0.001). Among respondents who do not perceive a high risk, a higher-than-expected proportion (33.3%) find personalized nutrition a particularly attractive option and a lower-than-expected proportion (17.6%) reject it. In contrast, those who perceive a high risk, reject the option at a higher rate than expected (44.8%) and accept it at a lower rate (13.8%).

The vast majority of respondents (78.8%) perceive that there is still a lot of uncertainty about personalized nutrition (perceived uncertainty), however, a significant—although weak—relationship in preferences (*χ*^2^(2) = 10.279, *p* = 0.006; Cramer’s *V* = 0.104; *p* = 0.006) can be noticed here, too, with very poor predictive power (Goodman–Kruskal tau = 0.004, *p* < 0.014; uncertainty coefficient = 0.005, *p* = 0.006). Those who believe there is still much uncertainty about the new technology reject it more than expected (32.4%) and find it an attractive option in a lower proportion than expected (21.4%), while those who do not feel much uncertainty show a higher rate of acceptance (30.7%) and a lower rate of rejection (23.3%).

In terms of subjective norms, we intended to examine whether individuals think people important to them would definitely switch to nutrigenomics-based personalized nutrition, and 45% of respondents claimed they would. In this case as well, a significant, moderately strong relationship (*χ*^2^(2) = 78.162, *p* < 0.001; Cramer’s *V* = 0.310, *p* < 0.001) was found between the subjective norm and the preferences, although the subjective norm is a very poor predictor (Goodman–Kruskal tau = 0.004, *p* < 0.014; uncertainty coefficient = 0.005, *p* = 0.006). Those with a stronger subjective norm find the option attractive in a higher proportion (37.3%) and reject it in a smaller proportion than expected (20.2%), while those who believe people important to them will not follow a personalized diet are more likely than expected to reject it (41.5%) and find it an attractive option to a lesser extent (13.2%).

When comparing the benefits and costs of genetically based personalized nutrition, 55.8% of respondents are more likely to see the benefits. The perceived benefit/cost, as a psychological process, has the strongest relationship to the preferences for genetic-based personalized nutrition (*χ*^2^(2)=127.687, *p* < 0.001; Cramer’s *V* = 0.376, *p* < 0.001), although it is also still weak as a predictor (Goodman–Kruskal tau = 0.063, *p* < 0.001; uncertainty coefficient = 0.068, *p* < 0.001). As expected, those who are more likely to see the benefits of the new technology consider the option more attractive than statistically expected (34.9%) and reject it to a lesser extent (16.2%), while for those who would rather consider the costs, it is less acceptable than expected (12%) and they reject it in a higher proportion (48.5%).

63.4% of the respondents claim that if genetic-based personalized nutrition is implemented in the future, they will be able to control their decision on adopting it, i.e. the majority of respondents can be characterized by strong perceived behavioural control. This behavioural control is also significantly—though weakly—related to the preferences for genetically based personalized nutrition (*χ*^2^(2) = 35.234, *p* < 0.001; Cramer’s *V* = 0.197, *p* < 0.001), with very poor predictive power (Goodman–Kruskal tau = 0.016, *p* < 0.001; uncertainty coefficient = 0.019, *p* < 0.001). One in three of those who perceive strong behavioural control were found to consider genetic test-based personalized nutrition particularly attractive, while only one in four of them rejects it. Among those who lack perceived behavioural control, the proportion of those considering genetic test-based personalized nutrition an attractive option is significantly lower than expected (14.5%), while the proportion of those rejecting the new technology is significantly higher than expected (40.4%).

## Discussion

The findings of our research suggest that in contrast to most previous research, the preference of Hungarian consumers for genetic-based personalized nutrition can be described as mixed. The proportion of respondents who consider the new option attractive and claim they would try it is only slightly lower (23.5%) than the results reported by Szakály et al. [52–54] (27%) based on their research conducted in 2014. The proportion of those in whom genetically based personalized nutrition evokes ambivalence (45.9%) has barely increased—by only 1.7 percentage points between 2014 and 2019—while the proportion of rejecters has increased by 1.8 percentage points to 30.6%. All this means that the consumer group that can be a potential target group for genetic-based personalized nutrition can be considered stable in the Hungarian market over time.

Similar to the findings of several previous research studies, certain socio-demographic variables significantly—although weakly—influence the preferences for genetic test-based personalized nutrition. As noted by Roosen et al. [47] and Stewart-Knox et al. [48], among Hungarian consumers, it is also women who are more likely to consider nutrigenomics-based personalized nutrition an attractive option. In contrast to the findings of Ahlgren et al. [43] and Stewart-Knox et al. [48], in our research, middle-aged people (40–49 years of age) are the least reluctant to use the new technology, while the older age group is more reluctant. In line with our expectations, confirming the findings of Cohen et al. [55] and Szakály et al. [52–54] it is those with a higher subjective income, who find nutrigenomics-based personalized nutrition more attractive and are less likely to reject it. In contrast to the results of Roosen et al. [46] and in accordance with the findings of Szakály et al. [52–54] the current research revealed that the educational level of Hungarian consumers is related to the rejection or acceptance of the new technology: those with a higher level of education accept it in a higher proportion and reject it the least, and vice versa.

Based on chi-square tests, it has also been established that all psychological characteristics significantly influence preferences for genetic-based personalized nutrition, and compared with the effect of socio-demographic criteria, on the whole, psychological processes (with the exception of perceived uncertainty) have a greater influence on the preference for genetic-based personalized nutrition. Our results indicate that it is perceived cost/benefit that is most strongly related to genetically based personalized dietary preferences, followed by perceived risk and subjective norms. Perceived uncertainty and perceived behavioural control, however, have only a weak relationship with genetic-based personalized dietary preferences. Previous research studies arrived at slightly different conclusions, i.e. the psychological mechanisms of perceived risk and uncertainty were found to be the primary factors influencing preferences for personalized nutrition, followed by perceived cost/benefit [53, 54]. The results of our research provide strong support for the framework developed by Ronteltap et al. [16, 61]; however, cost/benefit assessment appeared to be the most important construct in that research [61], followed by perceptions of the subjective norm. The results also confirm other previous research studies, primarily in the comparison of perceived benefits and perceived risks [56, 67]. The research findings outlined above confirms the hypothesis that personalized nutrition as a technology based on genetic testing is most attractive to consumers when it does have real added value and this benefit can also be perceived by consumers [16, 53, 54, 61]. The relative importance of subjective norms in turn suggests that the immediate environment, and important and credible opinion leaders influencing consumer decisions play a significant role in the process of accepting genetic-based personalized nutrition.

## Conclusions

Less than a quarter of Hungarian consumers consider the use of personalized nutrition based on genetic testing to be a particularly attractive option, which is a lower proportion than the one observed in 2014 [52–54]. It is an unfavourable process that the proportion of those who are insecure or dismissive about the new technology has increased—although only slightly—i.e. perceived uncertainty is currently stronger than in the previous period.

Compared with the magnitude of the effect of socio-demographic criteria, it can be concluded that, on the whole, psychological processes in the individual have a greater influence on the development of preferences for genetic-based personalized nutrition than any socio-demographic factor (with the exception of perceived uncertainty). This implies that everything individuals believe, think, know and expect from a personalized diet has a greater impact on their decisions than the demographic characteristics they can be described by. This also confirms the trend that there are more and more value-added products or value propositions (where a significant part of the value added is to be found in product innovation), for which psychological characteristics are/should be given more emphasis among the segmentation criteria.

Based on our results all the psychological processes incorporated in the model of Ronteltap et al. [61] could be accepted as factors with significant influencing power in preferences for genetically based personalized nutrition. However, since the multivariate analysis proved unsuccessful, their combined effects could not be examined, and consequently, in the present research, the assumption that the Ronteltap et al. [61] model would be valid without any changes among Hungarian consumers must be rejected. The results of our research are in line with most of the findings of Szakály et al. [52–54]; however, in our view, by transforming the measurement part of the model in the future, several factors may be found that can be involved in modelling and examining the cause-effect relations, since our results clearly demonstrate that there are a number of psychological processes in the development of genetic-based personalized dietary preferences.

The present research is limited by the level of the measurement scale of the applied variables. We believe that a multi-level scale would provide more opportunities for modelling as well as allow for validation and reliability studies of the model. However, since the dimensions of the model created by Ronteltap et al. [61] have a significant relationship with preferences for genetically based personalized nutrition, presumably, a transformation of the model would provide us with results that would allow a deeper exploration of the relationship between psychological characteristics and gene-based personalized nutrition.

## Supplementary Information

**Additional file 1: Table S1.** Items measuring the model constructs.

## Data Availability

The datasets used and/or analysed during the current study are available from the corresponding author on reasonable request.
