# Supplementary material for: Consumer acceptance of genetic-based personalized nutrition in Hungary
Source: Genes Nutr. 2021 Mar 1;16:3. doi: 10.1186/s12263-021-00683-7 (PMC7923598; doi:10.1186/s12263-021-00683-7)
Supplement: Supplementary file 1 — Additional file 1: Table S1. Items measuring the model constructs. [file 12263_2021_683_MOESM1_ESM.pdf]

Table S1. Items measuring the model constructs.

**1. Please choose one from the following options.**<br><br>*I think that a genetic test-based personalized nutrition is…*<br>1. a particularly attractive option, therefore I would use it.<br>2. both attractive and not attractive, with a view to preserving my health.<br>3. not an attractive option at all, therefore I would not use it.		
**2. Please rate the extent to which you agree with the following statements.**<br>*(1 – totally disagree, 5 – totally agree, 0 – do not know/do not answer)*		
1. Personalized nutrition enables me to live my life in good health for as long as possible.	1 – 2 – 3 – 4 – 5	0
2. Personalized nutrition enables us to delay the onset of disease so that a longer period of life be free from diseases.	1 – 2 – 3 – 4 – 5	0
3. It would be better if researchers fully agreed on the benefits of personalized nutrition.	1 – 2 – 3 – 4 – 5	0
4. It would be better if research further clarified the advantages and disadvantages of personalized nutrition.	1 – 2 – 3 – 4 – 5	0
5. It would be good if I could personally benefit from nutrition that fits my genetic background and thus be able to prevent diseases.	1 – 2 – 3 – 4 – 5	0
6. It would be good if researchers in particular would benefit from learning about the relationship between nutrition and genetics.	1 – 2 – 3 – 4 – 5	0
7. It would be good if the food industry in particular could benefit from being able to develop special foods by learning about the relationship between nutrition and genetics.	1 – 2 – 3 – 4 – 5	0
8. It is better if I can keep my old eating habits to the maximum and only need to supplement it with a few personalized products or dietary supplements.	1 – 2 – 3 – 4 – 5	0
9. It is better if I have to reshape my previous eating habits. There are some foods of which I will have to consume less and some of which I will have to consume more. I also need to add several new products to my diet.	1 – 2 – 3 – 4 – 5	0
10. It is better for me to be able to decide voluntarily whether to switch to genetically based personalized nutrition.	1 – 2 – 3 – 4 – 5	0
11. It would be better to make genetically based personalized nutrition mandatory for everyone.	1 – 2 – 3 – 4 – 5	0
**3. Please rate whether you agree with the following statements.**<br>*(1 – agree; 2 – disagree; 0 – do not know/do not answer)*		
1. If I consider all the pros and cons of genetically based personalized nutrition, I am positive about it.	1 – 2	0
2. I have the feeling that genetically based personalized nutrition brings about a lot of risks.	1 – 2	0
3. I feel that there is still a lot of uncertainty about genetically based personalized nutrition.	1 – 2	0
4. Most people who are important to me would be positive about switching to genetically based personalized nutrition.	1 – 2	0
5. If genetically based personalized nutrition were to materialize in the future, I will have full control over the decision to participate in it.	1 – 2	0
